# Statin Effects in Atrial Fibrillation-Related Stroke: A Systematic Review and Meta-Analysis

**DOI:** 10.3389/fneur.2020.589684

**Published:** 2020-10-09

**Authors:** Mi-Yeon Eun, Jin-Man Jung, Kang-Ho Choi, Woo-Keun Seo

**Affiliations:** ^1^Department of Neurology, Kyungpook National University Chilgok Hospital, School of Medicine, Kyungpook National University, Daegu, South Korea; ^2^Department of Neurology, Korea University Ansan Hospital, Korea University College of Medicine, Ansan, South Korea; ^3^Korea University Zebrafish Translational Medical Research Center, Ansan, South Korea; ^4^Department of Neurology, Chonnam National University Hospital, Gwangju, South Korea; ^5^Department of Neurology, Samsung Medical Center, Sungkyunkwan University School of Medicine, Seoul, South Korea

**Keywords:** atrial fibrillation, statins, stroke, mortality, functional outcome, systematic review, meta-analysis

## Abstract

**Background:** Statins lower the risk of recurrent stroke and mortality and improve outcomes in patients with ischemic stroke. However, the effects of statins on atrial fibrillation (AF)-related stroke are not well-established. Our study aims to investigate the effects of statin therapy on the clinical outcomes in patients with AF-related stroke.

**Methods:** Electronic databases (MEDLINE, Embase, and Scopus) were searched for previous studies on the effects of pre- and post-stroke statins on the clinical outcomes in AF-related stroke patients. The primary outcome was all-cause mortality. Secondary outcomes included recurrent ischemic stroke, acute coronary events, major adverse cardiovascular events (MACE), and short-term functional outcomes. We extracted hazard ratios (HRs) or odds ratios (ORs) with 95% confidence interval (CI) from each study and pooled them through a meta-analysis.

**Results:** A total of eight studies (five on post-stroke statins and three on pre-stroke statins) with 12,216 patients were included in the analysis. Post-stroke statin therapy reduced the risk of all-cause mortality (HR, 0.63; 95% CI, 0.55–0.74). This beneficial effect was sustained regardless of statin intensity. However, no significant associations were observed between statin therapy and a reduction in the risk of recurrent ischemic stroke, acute coronary events, or MACE. Pre-stroke statin use was associated with a lower risk of poor short-term functional outcomes (OR, 0.63; 95% CI, 0.47–0.85).

**Conclusions:** Statin therapy for AF-related stroke may reduce all-cause mortality and improve functional outcomes. Randomized controlled studies are warranted to confirm the effects of statins on the outcomes of AF-related stroke.

## Introduction

Statins or 3-hydroxy-3-methylglutaryl coenzyme-A reductase inhibitors reduce the risk of stroke, cardiovascular events, and mortality in patients with stroke (1, 2), and clinical practice guidelines strongly recommend statin therapy for atherosclerotic cardiovascular diseases and ischemic stroke (3). Statins decrease blood cholesterol and demonstrate pleiotropic effects such as anti-inflammation, endothelial nitric oxide synthase upregulation, and antithrombotic properties (4). Owing to these pleiotropic effects, statins may have broader therapeutic benefits, and they can be effective for non-atherosclerotic diseases. The beneficial effects of statins in treating non-atherosclerotic stroke, such as cancer-related stroke, cardioembolic stroke, and even hemorrhagic stroke, have been reported (5–7).

Atrial fibrillation (AF) is a major cause of ischemic stroke, and the prevalence of AF-related stroke has been increasing with the increase in life expectancy (8, 9). As patients with AF frequently have accompanying atherosclerotic risk factors, prescriptions of statins are also increasing (10, 11). However, the effects of statins in patients with AF-related stroke are not well-established. A major clinical trial, which demonstrated the effects of statins in patients with ischemic stroke, excluded patients with AF (1). Although several observational studies have reported that statins may be beneficial in patients with AF-related stroke, the results are inconclusive (12–14).

In this study, we aimed to investigate and summarize the effects of statin therapy on the clinical outcomes in patients with AF-related stroke through a systematic review and meta-analysis.

## Methods

### Search Strategy and Data Sources

We used comprehensive databases (MEDLINE, Embase, and Scopus) to search for previous studies that investigated the effects of pre- and post-stroke statin therapies on the clinical outcomes in patients with AF-related stroke. A literature search was performed by trained medical librarians (Eun Ju Lee and Eun-Ji Kang) from inception up to June 15th, 2020, using the following keywords and MeSH terms in MEDLINE: “atrial-fibrillation,” “stroke,” “statins,” “hydroxymethylglutaryl-CoA reductase inhibitors,” and “outcome.” The search strategy for MEDLINE was developed first, and it was then applied to the other databases. There was no restriction on language. Reference lists of the included articles and literature from manual searches were further screened for additional publications. This meta-analysis was conducted following the PRISMA guidelines (15). It was not registered on an international prospective registration site. This study was also exempt from approval from the Institutional Review Board of our institution because of its design (systematic review and meta-analysis).

### Study Selection

The following studies were considered eligible: (1) prospective or retrospective cohort studies or case-control studies; (2) studies that evaluated the effects of pre- or post-stroke statin treatments in patients with ischemic stroke and AF; and (3) studies reporting effect estimates using hazard ratios (HRs) or odds ratios (ORs) with a 95% confidence interval (CI) after statistical adjustment or matching for confounding factors. As we were interested in the effects of statin treatments in AF-related stroke, we excluded reports that evaluated the effects of statins in non-AF cardioembolic stroke. We, however, included one study that involved patients with cardioembolic stroke because it contained AF-related stroke data that could be obtained by contacting the author (6). If two or more studies reported overlapping data, we selected the one that described the time interval between statin therapy and ischemic stroke and data on the outcomes of interest. Two authors (Mi-Yeon Eun and Jin-Man Jung) independently selected the studies for meta-analysis and resolved any disagreements by consensus.

### Data Extraction and Assessment of Quality

The extracted data from the studies included the study period, design and setting, demographic findings, prescription rate of statins and anticoagulants, and hazard or odds ratios with 95% confidence interval for the clinical outcomes of interest. If necessary, we contacted the authors to obtain additional unpublished data. Quality assessments were performed using the Risk of Bias Assessment tool for Non-randomized Studies (RoBANS) (16). RoBANS comprises six components (selection of participants, confounding variables, measurement of intervention, blinding for outcome assessment, incomplete outcome data, and selective outcome reporting). A high, low, or unclear risk was assigned to each item using the assessment criteria. Data extraction and assessment of quality were performed independently by two authors (Mi-Yeon Eun and Jin-Man Jung), and any disagreements were resolved by discussion.

### Outcomes of Interest

The primary outcome of this study was all-cause mortality. We thought that it was appropriate to prove the pleiotropic effects of statins. Secondary outcomes included recurrent ischemic stroke, acute coronary events, major adverse cardiovascular events (MACE), and short-term poor functional outcomes. Acute coronary events included acute coronary syndrome and myocardial infarction according to the definitions used by the included studies. The MACE was defined following the definitions used in the included studies; such definitions are heterogeneous. A poor functional outcome was defined as mRS 4–6 or mRS 3–6 (from 1 month to 3 months after discharge) depending on the studies.

### Statistical Analysis

The meta-analysis in this study was performed using the outcomes reported by at least two of the included studies. We obtained the pooled effect sizes using the DerSimonian and Laird methods with the random-effects model. The meta-analyses of specific outcomes were pooled using HRs with 95% CI for post-stroke statin therapy and ORs with 95% CI for pre-stroke statin therapy from each study. We applied the Cochrane Q test and *I*^2^ statistics to estimate heterogeneity. An *I*^2^ value of <25% was considered low, >25% but <75% was considered moderate, and >75% was considered significant (17). Subgroup analyses of all-cause mortality and recurrent ischemic stroke were performed according to statin intensity (high and low-to-moderate) and the types of included patients (hospital-based and population-based). Sensitivity analyses were performed by sequential removal of an individual study to assess whether it influenced the overall results. Publication bias tests using funnel plot and Egger's test were conducted in cases where the included studies were at least ≥10. We used the RevMan software version 5.3 for these analyses.

## Results

### Study Characteristics

**Figure 1:**
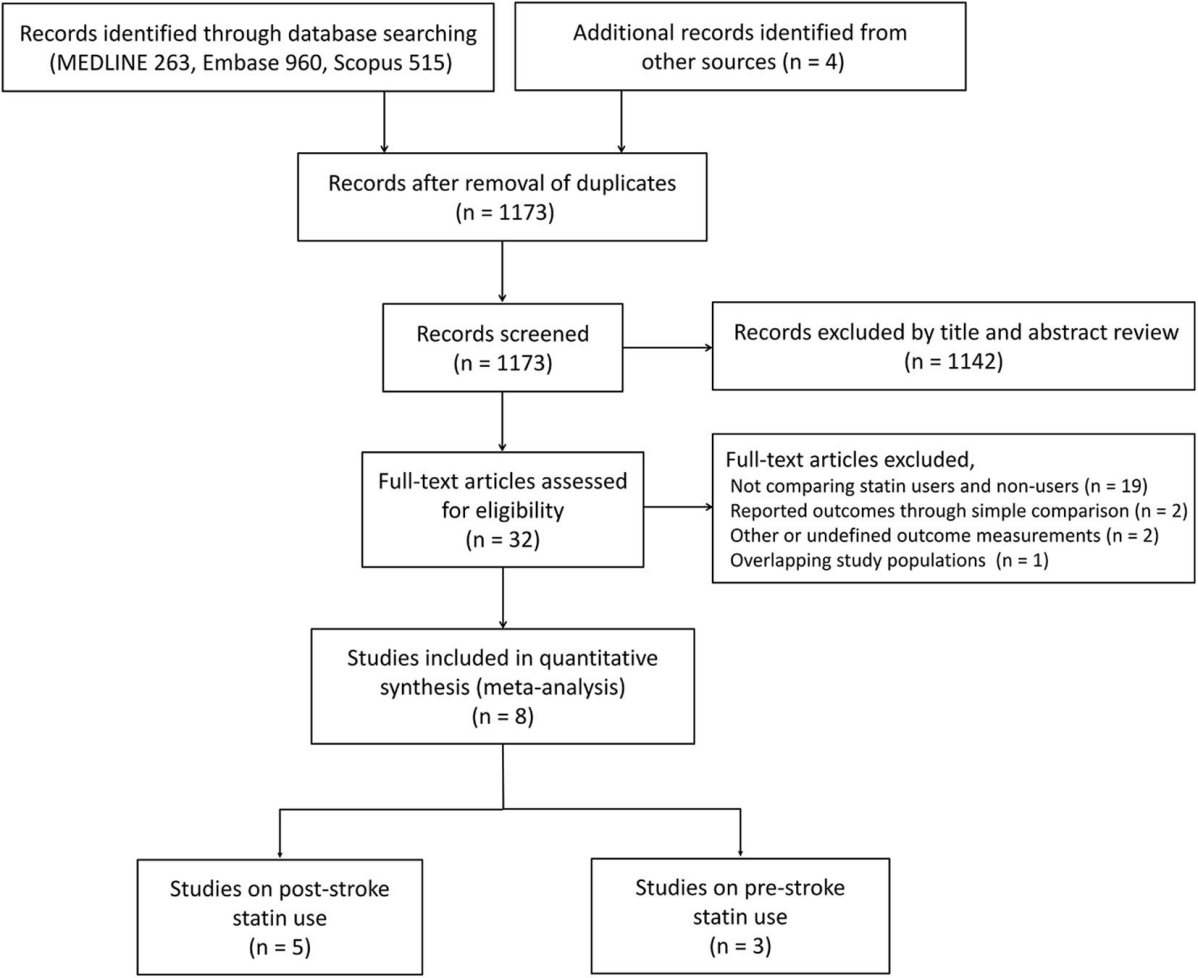
PRISMA flow diagram.

A total of 1,742 studies were obtained from the search. After removing duplicates and screening by title and abstract, we manually reviewed 32 full-text articles. Twenty-four articles were excluded for (1) not comparing statin users and non-users; (2) not reporting adjusted effect estimates; (3) not providing data about the outcomes of interest; and (4) having overlapping study populations. Finally, eight studies (five studies for post-stroke statin; three studies for pre-stroke statin) were included in this meta-analysis (Figure 1) (6, 12–14, 18–21). We excluded the study of Lin et al. (22) because of the population overlap with the study of Wu et al. (13) and the lack of information on the initiation time of statin treatment after AF-related stroke. The characteristics of the included studies are demonstrated in Table 1. The prevalence of paroxysmal AF ranged from 24.3 to 54.9% of the study population. The proportion of patients using post-stroke statin therapy in individual studies ranged from 25.2 to 73.3%. The percentage of patients managed with anticoagulants ranged from 23.6 to 71.3%. The longest median follow-up was 2.4 years. All of these publications were observational studies, and they included 12,216 subjects. Six studies were performed using a single-center or multicenter hospital-based registry on acute ischemic stroke (6, 12, 14, 19–21). The other two studies were population-based (13, 18). The adjusted hazard or odds ratios were obtained from these studies. The common adjusted variables for the effect of post-stroke statin on mortality in hospital-based studies were age, sex, hypertension, diabetes mellitus, and stroke severity assessed by the National Institute of Health Stroke Scale (NIHSS). Hayden et al. (18) conducted a population-based cohort study, which adjusted pre-stroke functional status, type of AF, and CHADS_2_ score for assessing the effect of statins on mortality (18). A study by Wu et al. (13) identified subjects using the Taiwan National Health Insurance Research Database. This study included patients receiving statins after recent ischemic stroke and matched controls in a 1:2 ratio based on age, sex, cardiovascular risk factors, and the estimated NIHSS score.

**Table 1:** Characteristics of included studies.

**Author**	**Study period**	**Design/setting**	**No**	**Baseline features**						**Outcome**		**Follow-up (IQR)**
				**Age (SD)**	**Male**	**HTN**	**Hyperlipidemia**	**Statin**	**OAC**	**Primary**	**Secondary**	
Ntaios G^*^	2000–2011	(Retrospective/ 11 centers	404	67.2 (12.3)	68.2	76.0	33.7	25.2	71.3	Mortality	Recurrent IS MACE^†^	22 mon (9–48)
Choi JY^*^	2008–2012	Retrospective/ 3 centers	376	71.2(10.9)	52.4	69.2	N/A	45.5	64.6	Mortality Recurrent IS	None	17.4 mon(8.0–34.1)
Hayden DT^*^	2005–2006	Prospective/ Population-based cohort	160	N/A	N/A	N/A	N/A	72.7	36.5	Mortality	None	N/A
Choi KH^*^^†^	2013–2015	Prospective/ 11 centers	2,153	73.2 (9.8)	52.0	69.9	23.3	73.3	62.5	MACE^‡^	Components of MACE^‡^ Functional outcome	17.3 mon(4.3–31.3)
Wu YL^*^	2000–2011	Retrospective/ Multicenter	4,638	75.6 (7.4)	49.1	96.6	N/A	33.3	39.7	Recurrent stroke	Mortality Recurrent IS MACE^§^ MI	2.4 years(1.3–4.1)
Ko D^†^	2000–2011	Retrospective/ 3 centers	1,030	77.0 (11.1)	44.1	90.7	N/A	38.8 (pre-)	28.4	Poor functional outcome (mRS 4–6)	None	30 days
He L^†^	2015–2018	Prospective/4 centers	242	N/A	48.8	69.8	49.2	43.8 (pre-)	23.6	Mortality Poor functional outcome (mRS 3–6)	Composite of mortality/major disability	3 mon
Song TJ^†^	2013–2015	Prospective/ 11 centers	3,213	73.4 (9.6)	50.7	68.7	18.6	20.2 (pre-) 70.0 (post-)	53.7	Poor functional outcome (mRS 3–6)	None	3 mon

*SD, standard deviation; IQR, interquartile range; OAC, oral anticoagulation; N/A, not applicable; IS, ischemic stroke; MACE, major adverse cardiovascular events; mon, months; MI, myocardial infarction; mRS, modified Rankin Scale; HTN, hypertension*.

* *Post-stroke statin study*.

† *Pre-stroke statin study*.

*MACE^†^ was defined as stroke recurrence, myocardial infarction, aneurysm rupture, and sudden cardiac death*.

MACE

‡ * was defined as death from any cause, non-fatal stroke, acute coronary syndrome, and major hemorrhage*.

MACE

§ * was defined as any ischemic or hemorrhagic stroke, and myocardial infarction*.

### Quality of the Included Studies

Potential sources of bias in the included studies are presented in Supplementary Figure 1. The likelihood of selection bias was generally low except for some studies on pre-stroke statin effects (20, 21). As we included studies that reported estimates after adjusting for or matching confounding factors, the risk of confounding was also low. Blinding was not applied to the outcome assessments in any of the included studies. However, mortality was considered unaffected by blinding. Reporting bias was unclear in most of these publications.

### Effect of Post-stroke Statin Therapy on Clinical Outcomes

**Figure 2:**
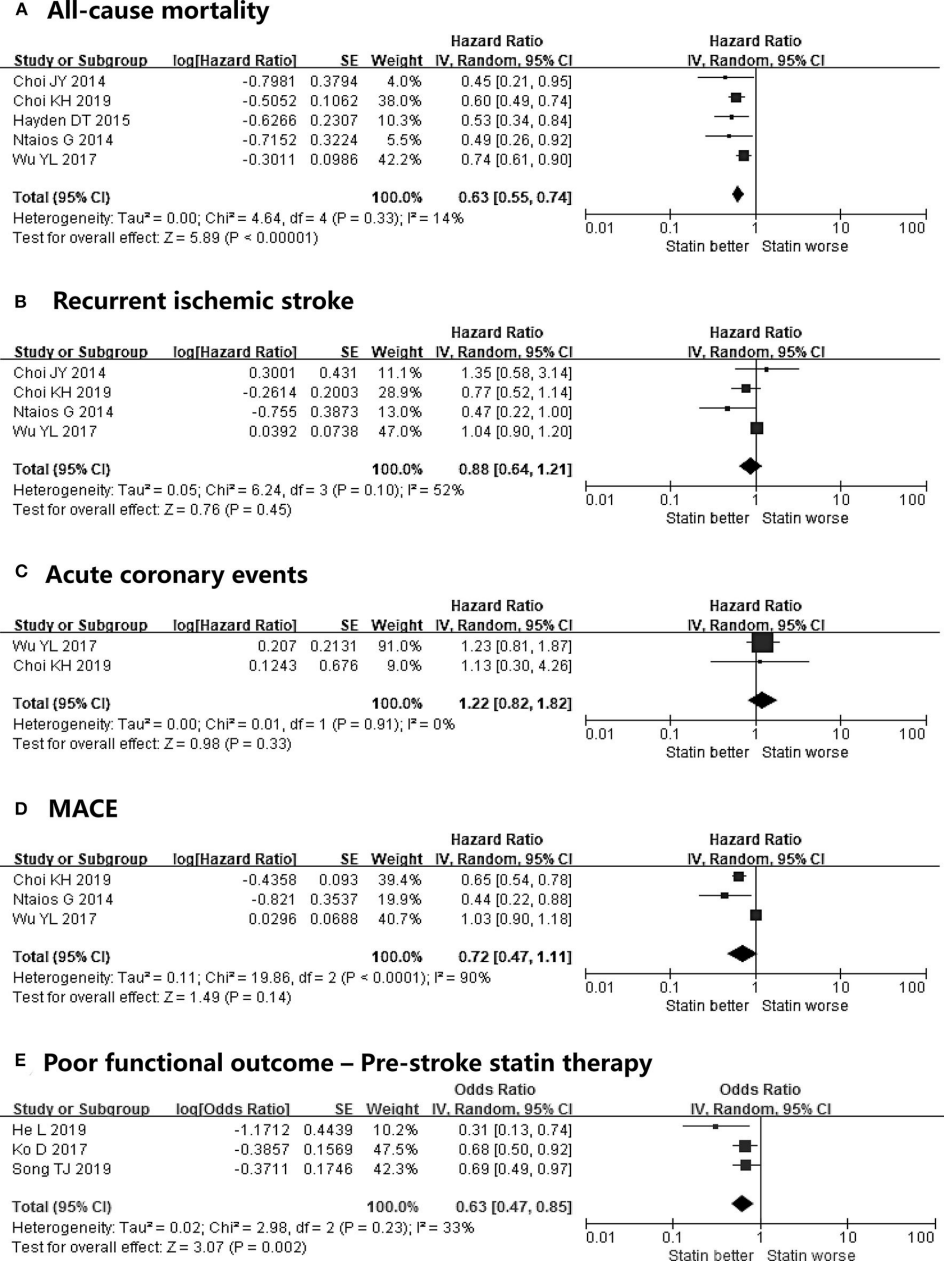
Meta-analyses on the effect of post-stroke statin therapy on **(A)** all-cause mortality, **(B)** recurrent ischemic stroke, **(C)** acute coronary events, **(D)** MACE, and meta-analysis on the effects of pre-stroke statin therapy on **(E)** poor functional outcomes in patients with atrial fibrillation-related stroke. MACE, major adverse cardiovascular events.

The number of included studies in the meta-analyses on all-cause mortality, recurrent ischemic stroke, acute coronary events, and MACE were 5, 4, 2, and 3, respectively (Figure 2). Post-stroke statin therapy was associated with a reduced risk of all-cause mortality (HR, 0.63; 95% CI, 0.55–0.74) with a low heterogeneity (*I*^2^ = 14%). The effect of statins on mortality was maintained even after adjustment for the type of AF (HR, 0.53; 95% CI, 0.34–0.84) in a study by Hayden et al. (18). In contrast, post-stroke statins were not effective in preventing recurrent ischemic stroke (HR, 0.88; 95% CI, 0.64–1.21) and acute coronary events (HR, 1.22; 95% CI, 0.82–1.82). The results for recurrent ischemic stroke showed moderate heterogeneity between the studies (*I*^2^ = 52%). On the other hand, low heterogeneity (*I*^2^ = 0%) was found for acute coronary events. Regarding MACE, post-stroke statin therapy was not associated with a decreased risk of events (HR, 0.72; 95% CI, 0.47–1.11) with high heterogeneity (*I*^2^ = 90%).

### Subgroup Analyses of Post-stroke Statin Therapy Studies

**Figure 3:**
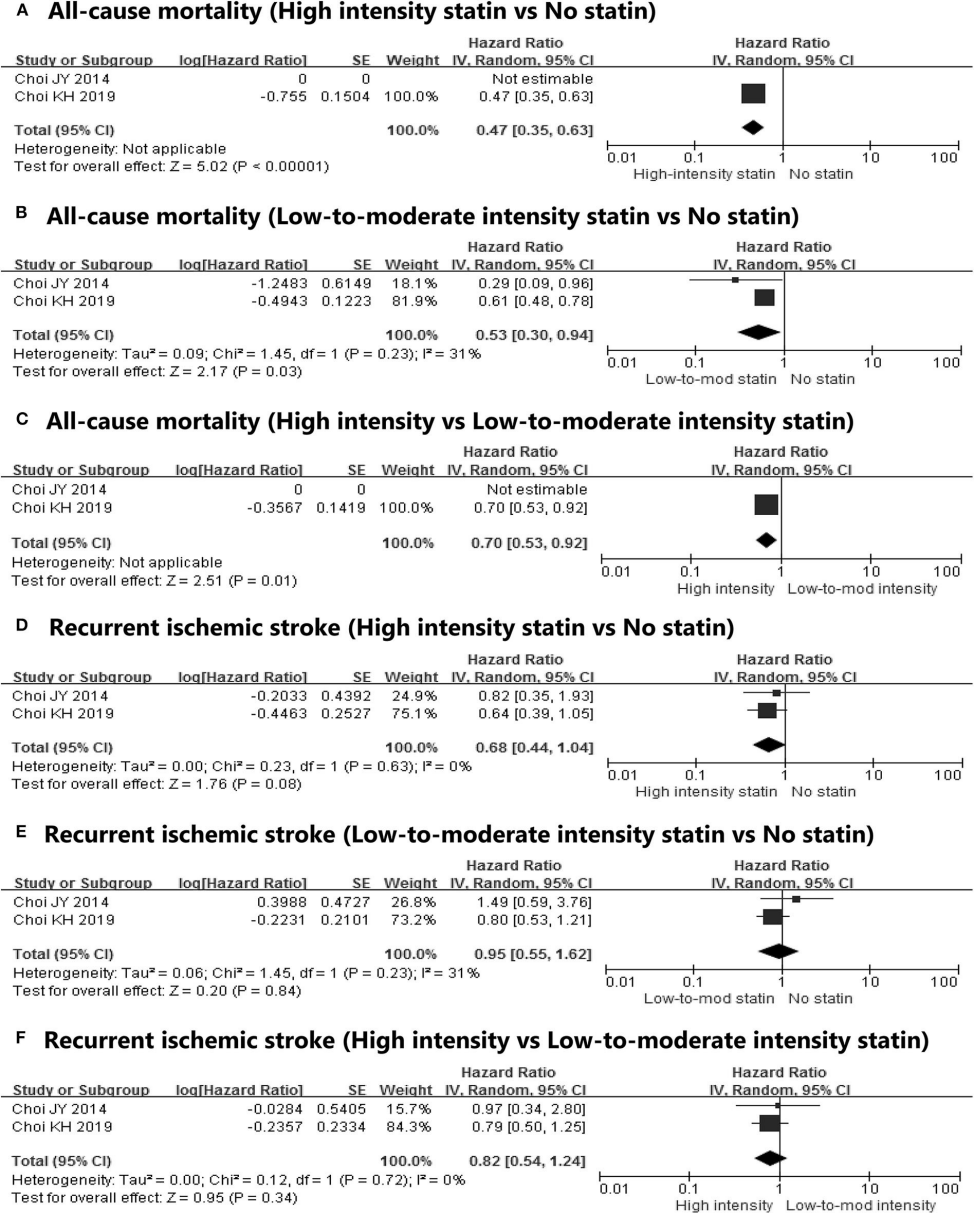
Subgroup meta-analyses on the effect of post-statin therapy on **(A)** all-cause mortality (high-intensity statin vs. no statin), **(B)** all-cause mortality (low-to-intermediate-intensity statin vs. no statin), **(C)** all-cause mortality (high-intensity vs. low-to-moderate intensity statin), **(D)** recurrent ischemic stroke (high-intensity statin vs. no statin), **(E)** recurrent ischemic stroke (low-to-intermediate-intensity statin vs. no statin), and **(F)** recurrent ischemic stroke (high-intensity vs. low-to-moderate-intensity statin) stratified by the intensity of statins.

We conducted subgroup analyses to assess the effects of post-stroke statin therapy on clinical outcomes stratified by statin intensity and patient subgroup type (hospital-based vs. population-based). Two studies analyzed the intensity-related effects of statin therapy on mortality and recurrent ischemic stroke (6, 14). In terms of all-cause mortality, a meta-analysis of the high-intensity vs. no statin and the high-intensity vs. low-to-moderate-intensity statin groups could not be conducted because no deaths occurred in the high-intensity group in one study (6) during the follow-up period (Figures 3A,C). In the other report, the risk of all-cause mortality was lower with high-intensity statin therapy than without statin therapy (HR, 0.47; 95% CI, 0.35–0.63), and it was more effective than the low-to-intermediate intensity statin treatments (HR, 0.70; 95% CI, 0.53–0.92) (14). The risk of all-cause mortality was also lower with low-to-moderate intensity statin therapy in our meta-analysis than without statin therapy with moderate heterogeneity (HR, 0.53; 95% CI, 0.30–0.94) (Figure 3B). The prevention of recurrent ischemic stroke by statin therapy was not effective irrespective of the intensity (Figures 3D,E). However, high-intensity statin therapies marginally decreased the risk of recurrent ischemic stroke (HR, 0.68; 95% CI, 0.44–1.04) (Figure 3D). High-intensity statin therapy was not more effective in lowering the risk of recurrent ischemic stroke than low-to-moderate intensity statin therapy (Figure 3F).

The subgroup analyses showed that post-stroke statin therapy was associated with a reduced risk of mortality in the hospital-based (OR, 0.58; 95% CI, 0.48–0.70) and population-based studies (OR 0.67; 95% CI 0.49–0.90) with low heterogeneity. However, the post-stroke statin therapy had no benefit for the prevention of recurrent ischemic stroke in subgroup analyses, and these results were consistent with the primary meta-analysis. Regarding MACE, studies using a hospital-based registry demonstrated favorable results in post-stroke statin users (OR, 0.62; 95% CI, 0.48–0.79); this result was not seen in the population-based studies (OR 1.03; 95% CI, 0.90–1.18). The outcomes of the subgroup analyses, stratified by the patient subgroup type, are shown in Supplementary Figure 2.

### Sensitivity Analysis and Publication Bias

We conducted a sensitivity analysis of post-stroke statin effects on all-cause mortality. It demonstrated that the exclusion of any individual study did not significantly influence the effect of post-stroke statin therapy (Supplementary Figure 3). We did not use Egger's test and the funnel plot; we planned to use them if at least ten studies were included in the meta-analysis.

### Effect of Pre-stroke Statin Therapy on Clinical Outcomes

Three studies were used in our meta-analysis for poor functional outcomes (19–21). Pre-stroke statin therapy was associated with a reduced risk of poor functional outcomes (OR, 0.63; 95% CI, 0.47–0.85) with moderate heterogeneity (*I*^2^ = 33%) (Figure 2E). Among the included studies, only the study by He et al. (19) adjusted the type of AF. Although a meta-analysis was not conducted, the study by He et al. (19) showed that pre-stroke statin was inversely associated with the 3-month mortality (OR, 0.38; 95% CI, 0.16–0.91). We did not perform subgroup and sensitivity analyses owing to the limited number of studies included.

## Discussion

With data of 12,216 patients from eight studies, this meta-analysis demonstrates the beneficial effect of pre- and post-stroke statin therapy in patients with AF-related stroke. Specifically, post-stroke statin therapy was associated with a 36% reduced risk of all-cause mortality. The result was consistent across the included studies with low heterogeneity. In addition, it was irrespective of statin intensity and patient subgroup type. Although a meta-analysis was not performed, high-intensity statin therapy seemed to be associated with a lower risk of all-cause mortality than low-to-moderate-intensity statin therapy (14). However, post-stroke statin therapy in those studies did not prevent recurrent ischemic stroke, acute coronary events, and MACE. On the other hand, pre-stroke statin therapy was associated with a lower risk of short-term poor functional outcomes.

Several studies have reported that statin therapy is beneficial in patients with stroke. The Stroke Prevention by Aggressive Reduction in Cholesterol Levels (SPARCL) trial reduced the incidence of strokes and cardiovascular events. However, cardioembolic stroke was excluded in the SPARCL trial (1). The North Dublin Population Stroke Study and observational study from the Athenian Stroke Registry, which included patients with AF, revealed that post-stroke statin therapy reduced mortality (23, 24). However, there was no high-level evidence on the effect of statins in patients with AF-related non-cardioembolic stroke. Our meta-analysis verified that statins had potential benefits even in cases restricted to AF-related stroke irrespective of the time of initiation of statin administration.

Notably, in our meta-analysis, post-stroke statin therapy was significantly associated with a lower risk of mortality in AF-related stroke. Moreover, pre-stroke statin usage was likely to reduce mortality. AF shares risk factors with atherosclerosis, which include old age, hypertension, and diabetes mellitus (25). As statins have anti-atherogenic and antithrombotic effects, they may reduce the risk of vascular events and cardiovascular mortality in AF-related stroke (26). However, in this meta-analysis, statin therapy did not reduce the vascular events, including recurrent ischemic stroke, acute coronary events, and MACE. Recent cohort studies involving older adult participants without atherosclerotic cardiovascular disease have reported that statin treatment has a major advantage for mortality over prevention of ischemic stroke or myocardial infarction (27).

The mechanism by which statins reduce stroke-related mortality is uncertain. Several biomarkers of stroke indicate that various mechanisms underlie stroke occurrence and prognosis (28, 29). We speculated that the systemic pleiotropic effects of statins, including an anti-inflammatory effect, anti-oxidant effect, improvement in endothelial function, and angiogenesis, may contribute to reduced mortality (4, 30). Additionally, post-stroke statins were associated with good functional outcomes in patients with stroke because of the neuroprotective effects (2, 31). Since good functional outcomes after stroke were independently predictive of survival, statins may have favorable effects on mortality (32). Finally, statins have potential benefits for heart disorders, which are major causes of mortality in patients with ischemic stroke. Anti-inflammatory and anti-oxidant effects of statins may result in an anti-arrhythmic potential and reduce the burden of AF (33), and statins have been reported to prevent the recurrence of AF, especially in patients with coronary artery disease (34). Besides, a recent cohort study demonstrated that statin therapy also contributed to reduced mortality and hospitalization in patients with heart failure with preserved ejection fraction (35).

Although a meta-analysis was not performed, high-intensity statins seemed to be more effective in reducing all-cause mortality than low-to-moderate-intensity statins in one of the included studies (14). Previous studies involving patients with acute ischemic stroke or atherosclerotic cardiovascular disease demonstrated an association between the intensity of statin therapy and mortality (36, 37). However, there was insufficient evidence to confirm this hypothesis because there were only a few relevant studies on AF-related stroke.

In our meta-analysis, post-stroke statin therapy was not effective in preventing recurrent ischemic stroke in patients with AF-related stroke. Anticoagulation is the main treatment for prevention of stroke or systemic embolization due to AF. Although statins are presumed to have antithrombotic effects (4), these effects may not be sufficient to prevent recurrent ischemic stroke caused by AF. These results were consistent with the results of a recent observational study involving patients with cardioembolic stroke that indicated that post-stroke statin therapy was associated with a reduced risk of mortality but not associated with the risk of recurrent stroke (38).

However, post-stroke statins can lower the risk of ischemic stroke of atherosclerotic origin. In a study by Ntaios et al. (12) post-stroke statins appeared to decrease the risk of recurrent stroke. Patients included in this study had relatively frequent hypertension (76.0%) and high low-density lipoprotein cholesterol levels [(statin user, 133.1 mg/dl (47.7); non-statin user, 117.5 mg/dl (37.0)] in post-stroke statin studies. Besides, the proportion of patients receiving anticoagulation was the highest (71.3%). The effect size of statins in preventing atherosclerotic vascular events may vary depending on the risk factors. Furthermore, these effects can be prominent in patients undergoing anticoagulation (39). Thus, baseline characteristics and anticoagulation status should be considered to interpret the effects of statin therapy in AF-related stroke.

Statin therapy was also not effective for the prevention of MACE. However, there was significant heterogeneity among the studies because of the varying definitions of MACE. In two studies that included mortality in the definition of MACE, post-stroke statin therapy was effective in reducing MACE (12, 14). These findings also supported the possible beneficial effects of statins on the mortality observed in our study.

In our meta-analysis, pre-stroke statin therapy was related to the reduced risk of short-term poor functional outcomes in AF-related stroke. These results were consistent with reports from previous studies that indicated pre-stroke statins to be associated with milder stroke and improved functional outcomes (40, 41). Statins can help reverse endothelial dysfunction, augmentation in the nitric oxide-mediated vasodilation, or plaque stabilization. Furthermore, they were reported to increase the collateral blood flow in AF-related stroke, leading to a lower infarct core and more efficient recoveries (42). Thus, pre-stroke statins were presumed to be neuroprotective against AF-related as well as atherosclerotic stroke.

Several limitations should be noted. First, we were unable to exclude the possibility of undetected confounding and selection bias because all of our included reports were non-randomized observational studies. Most of the included studies also had a retrospective design. Second, the baseline characteristics and the definitions of the outcomes in the included studies were heterogeneous. Thus, some results of these meta-analyses also had moderate to high statistical heterogeneity. Third, the effects of pre-stroke and post-stroke statin therapies overlap and do not occur in isolation. Most patients treated with pre-stroke statin therapy continued statin therapy after stroke. A considerable number of patients receiving post-stroke statin therapy also received pre-stroke statin therapy. Finally, our meta-analysis was not an individual patient-level pooled analysis, and therefore, we could not fully evaluate the statin type, dosage, adherence, the oral anticoagulant type at discharge, the subtype of AF such as paroxysmal vs. sustained, valvular vs. non-valvular, and the co-existence of a mechanical heart valve.

Despite these limitations, our meta-analysis has some strengths. First, we focused on the effects of statins in patients with AF-related, and not cardioembolic, stroke. Since cardioembolic stroke is attributable to heterogeneous etiologies, the effect of statins may differ with the various causes. Second, we obtained and pooled the adjusted effect estimates to reduce the confounding effects of other vascular risk factors. It is important because the therapeutic effects of statins may vary with comorbid vascular risk factors. Consequently, our meta-analysis demonstrated that the effects of statins were not limited to atherosclerotic stroke. Statins showed favorable effects on mortality and functional outcomes in patients with AF-related stroke even after the adjustment of atherosclerotic vascular risk factors. Therefore, we suggest that statins may be beneficial in patients with AF-related stroke, irrespective of atherosclerotic risk factors.

## Conclusions

Statin therapy is associated with a reduced risk of all-cause mortality, and pre-stroke statins can improve functional outcomes in AF-related stroke. Given the limitations of this study, especially related to the observational studies included, well-designed randomized controlled studies are required to confirm the effects of statins on the clinical outcomes of AF-related stroke.

## Data Availability Statement

All datasets generated for this study are included in the article/Supplementary Material.

## Author Contributions

M-YE searched for studies, screened references, extracted data, assessed risk of bias, performed the analyses, and drafted the manuscript. J-MJ conceived the study, searched for studies, screened references, collected data, assessed risk of bias, performed the analyses, drafted, and critically revised the manuscript. K-HC contributed to data collection and critically revised the manuscript. W-KS contributed to data collection and edited and critically revised the manuscript. All authors contributed to the article and approved the submitted version.

## Conflict of Interest

J-MJ has received lecture honoraria from Pfizer, Sanofi-Aventis, Otsuka, Dong-A, Hanmi Pharmaceutical Co., Ltd., and Boryung Pharmaceutical Co., Ltd.; a study grant from Il-dong and Cheiljedang Pharmaceutical Co., Ltd.; consulting fees from OBELAB Inc and Daewoong Pharmaceutical Co., Ltd. W-KS received honoraria for lectures from Pfizer, Sanofi-Aventis, Otsuka Korea, Dong-A Pharmaceutical Co., Ltd., Beyer, Daewoong Pharmaceutical Co., Ltd., Daiichi Sankyo Korea Co., Ltd., and Boryung Pharmaceutical Co., Ltd.; a study grant from Daiichi Sankyo Korea Co., Ltd.; consulting fees from OBELAB Inc. The remaining authors declare that the research was conducted without any commercial or financial relationships that could be construed as potential conflicts of interest.
