# Supplementary material for: Statin Effects in Atrial Fibrillation-Related Stroke: A Systematic Review and Meta-Analysis
Source: Front Neurol. 2020 Oct 9;11:589684. doi: 10.3389/fneur.2020.589684 (PMC7581731; doi:10.3389/fneur.2020.589684)
Supplement: Supplementary file 1 [file Table_1.DOCX]

Supplementary Material

# Supplementary Figure 1. Risk of bias depicted by colors (red: high-risk; green: low-risk; yellow: unclear)

**Supplementary Figure 2.** Meta-analyses of the effects of post-statin therapy on clinical outcomes stratified by the patient subgroup type (hospital-based vs. population-based)

1. All-cause mortality
2. Recurrent ischemic stroke
3. MACE

MACE, major adverse cardiovascular events

**Supplementary Figure 3.** Sensitivity analyses of studies on post-stroke statin effects on all-cause mortality

**Supplementary Figure 1.** Risk of bias depicted by colors (red: high-risk; green: low-risk; yellow: unclear)

**
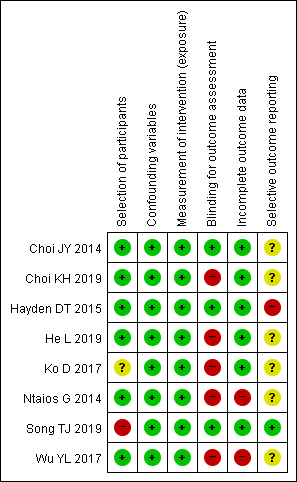
**

**Supplementary Figure 2.** Meta-analyses of the effects of post-statin therapy on clinical outcomes stratified by the patient subgroup type (hospital-based vs. population-based)

(A) All-cause mortality

##
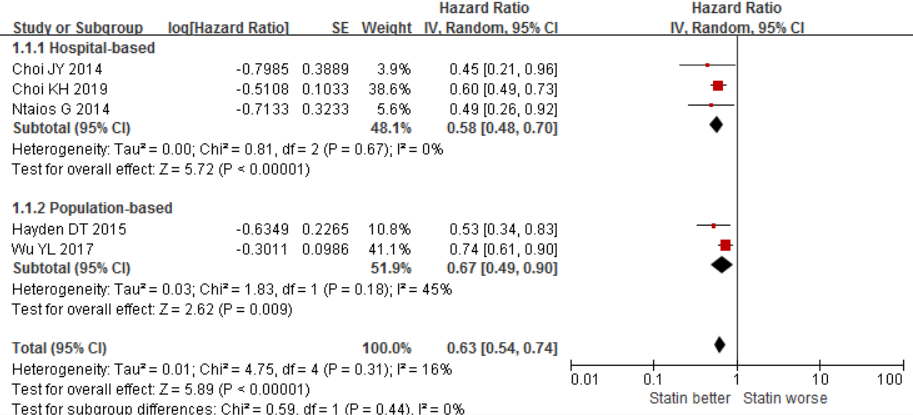


(B) Recurrent ischemic stroke **
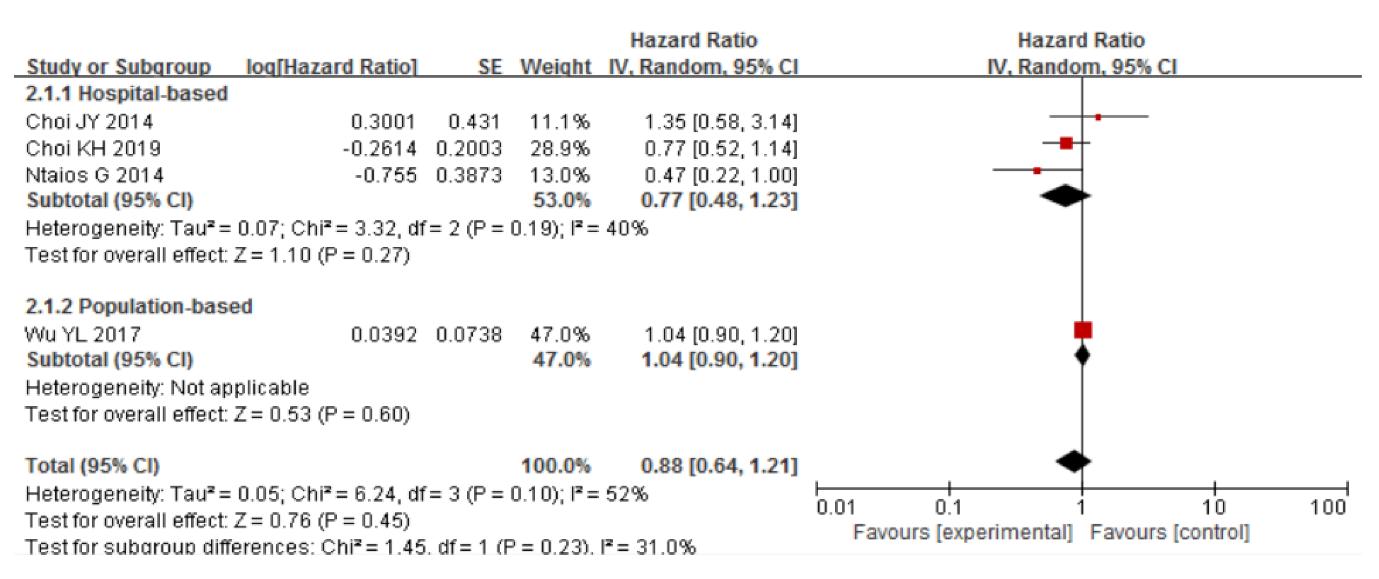
**

(C) MACE

**
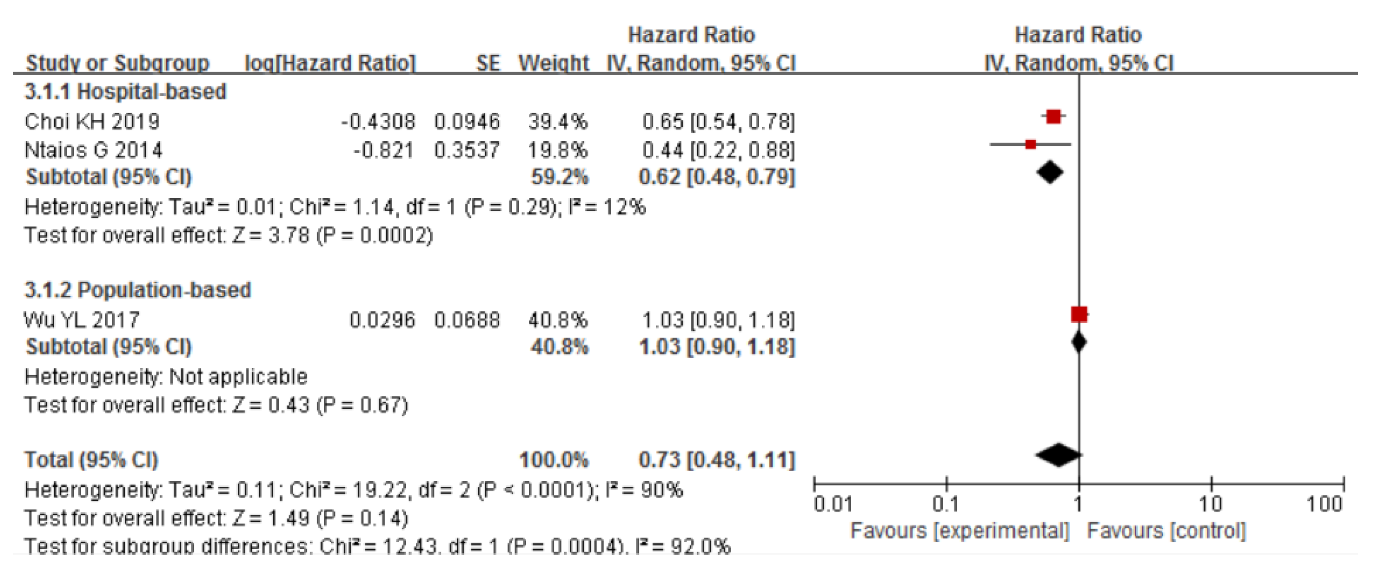
**

**Supplementary Figure 3.** Sensitivity analyses of studies on post-stroke statin effects on all-cause mortality

##
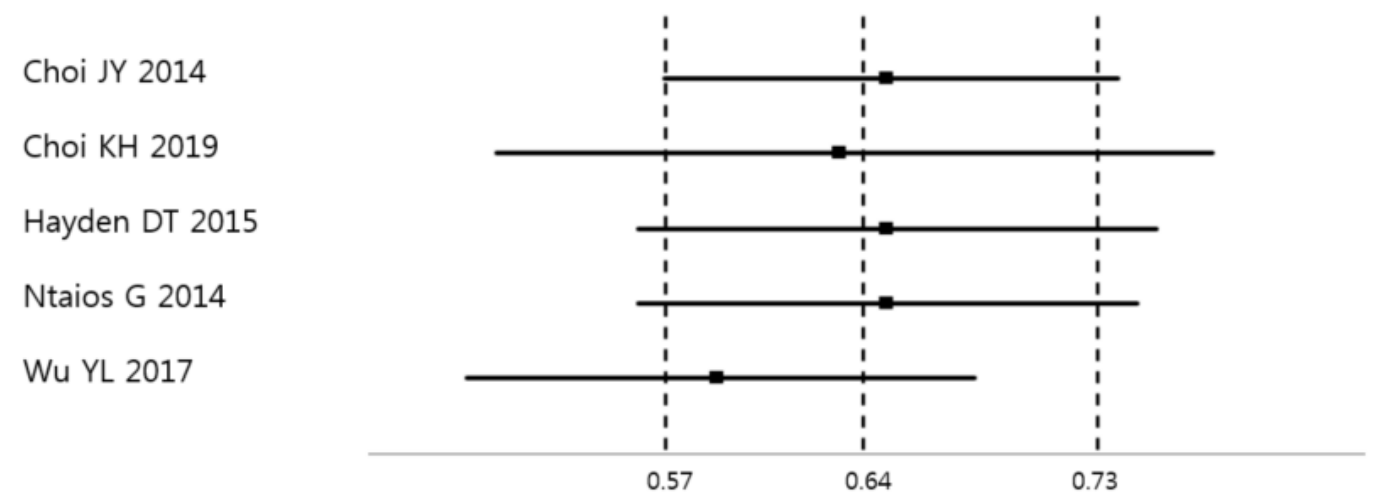


Each horizontal line indicates a repeated result from the pooled analysis after sequentially excluding the corresponding study. Vertical dotted lines represent pooled estimates and 95% confidence intervals (CIs) using all data from six, five, or nine studies, respectively.
